# Non-Invasive Electrochemical Biosensors Operating in Human Physiological Fluids

**DOI:** 10.3390/s20216352

**Published:** 2020-11-07

**Authors:** Magnus Falk, Carolin Psotta, Stefan Cirovic, Sergey Shleev

**Affiliations:** 1 Department of Biomedical Science, Faculty of Health and Society, and Biofilms—Research Center for Biointerfaces, Malmö University, 20506 Malmö, Sweden; magnus.falk@mau.se (M.F.); carolin.psotta@mau.se (C.P.); stefan.cirovic@mau.se (S.C.); 2 Aptusens AB, 293 94 Kyrkhult, Sweden

**Keywords:** non-invasive biosensors, human physiological fluids, tears, sweat, saliva, urine

## Abstract

Non-invasive healthcare technologies are an important part of research and development nowadays due to the low cost and convenience offered to both healthcare receivers and providers. This work overviews the recent advances in the field of non-invasive electrochemical biosensors operating in secreted human physiological fluids, viz. tears, sweat, saliva, and urine. Described electrochemical devices are based on different electrochemical techniques, viz. amperometry, coulometry, cyclic voltammetry, and impedance spectroscopy. Challenges that confront researchers in this exciting area and key requirements for biodevices are discussed. It is concluded that the field of non-invasive sensing of biomarkers in bodily fluid is highly convoluted. Nonetheless, if the drawbacks are appropriately addressed, and the pitfalls are adroitly circumvented, the approach will most certainly disrupt current clinical and self-monitoring practices.

## 1. Introduction

Owing to the low cost and convenience shared by healthcare receivers and providers alike, non-invasive healthcare technologies have become increasingly important parts of current research and development [1]. Non-invasive measurements include the use of sweat [2], urine [3], saliva [4], and tears [5], but can also rely on fluid-free technologies. The last option is more attractive because of fast and inexpensive analyses with convenient fluid independent procedures, and the concerns in the scientific community regarding correlations between bioanalyte concentration in blood and in other physiological fluids can be disregarded [6,7,8,9]. In addition to traditional fluid-free non-invasive technologies, which are known and have been used for ages, e.g., electrocardiography [10,11], many other non-invasive instruments have been developed, such as cardiovascular diagnostic systems [12], bioimpedance based scales [13,14], and even non-invasive blood analyzers to measure sentinel substances in blood, e.g., hemoglobin [15], oxygen [16], and glucose [17,18]. Hence, fluid-free oximeters are relied on and widely used in acute and critical care [19], but other fluid-free non-invasive blood analyzers, e.g., glucometers and hemoglobinometers, are far from accurate and the readings cannot be trusted [20,21,22]. Drawing on the contentious performance of current fluid-free analyzers, and since many important bioanalytes cannot be measured using fluid-free technologies, this review is focused on fluid-based biosensors operating in different physiological fluids, viz. tears, saliva, sweat, and urine. Among the variety of fluid-based non-invasive biosensors, this review is focused on electrochemical biodevices.

For all electrochemical techniques, the most common electrode materials are silver, gold, platinum, and carbon, e.g., graphene, graphite, carbon nanotubes, and glassy carbon [23]. Regarding carbon, the working electrode area can be tweaked by selecting from the carbon alternatives [24], and in general, the performance and sensing abilities and can be improved by surface modification using e.g., polymers, nanofibers, or nanoparticles [23,24]. Biomolecules are immobilized on the working area of the electrode surface, and act as recognition elements to generate and transduce an output signal. The selectivity and sensitivity of a particular recognition element can be finely tuned by appropriate helper elements [23,25]. The main components of a complete biosensor for detection of analytes in bodily fluids are the following: bioreceptor, transducer, electronics, and display.

A variety of compounds of clinical relevance are present in secreted physiological fluids, most of which can be converted by different oxidoreductases, and many by oxidases [5,6,7,9,10,11]. Hydrogen peroxide (H_2_O_2_), a common by-product of oxidases, is used as the terminal element in most enzyme-based biosensors and analytical kits, and H_2_O_2_ can be assessed to estimate the concentration of analytes. In a review, it is impossible to cover all electrochemical techniques used for the detection of analytes in human physiological fluids, and it is equally impossible to even briefly describe all biosensors operating in urine, sweat, sweat, and saliva. Below, we will describe a few examples of a rich variety of biodevices that rely on amperometry, coulometry, cyclic voltammetry, and impedance spectroscopy.

However, prior to the description, the following should be emphasised. On the one hand, exuded human physiological fluids are well suited as information vehicles and sources, provided that the available data can be sampled reliably. Tears, sweat, saliva, and urine are known to carry substances indicative of the health status of an individual. Glucose, lactate, ascorbate, urea, creatinine, as well as their metabolites, and hormones and their metabolites, are all examples of species present in human physiological fluids. By assaying amounts or relative amounts, or, by gauging appearance or disappearance rates, a reasonably accurate appreciation of the health status of an individual can be made. On the other hand, when it comes to non-invasive analysis of bioanalytes in secreted/exuded physiological fluids for diagnostics, it seems pertinent to highlight a major ambiguity, which attenuates the enthusiasm of medical doctors and in general seriously alerts the biomedical community. Unfortunately, data dispersion in the literature, when it comes to amounts, and sometimes even the presence of bioanalytes in physiological fluids, borders on the ridiculous. One can find all possible reports, from no correlation, via certain correlation, up to direct straight dependences between concentrations in different physiological fluids. Neither direct nor indirect reasons for this will be discussed in the current review because of the lack of space and possible loss of focus. It is obvious, however, that the nonsensical dispersion of data definitely calls for proper (i.e., with adequate sampling, taking into account differences in basal, induced, emotional fluids, as well as selective and appropriately sensitive determination, using modern techniques, in full control of possible pitfalls) chemical analysis of main bioanalytes in human physiological fluids naturally released outside the body and simultaneous comparison of blood concentrations.

## 2. Fluid-Based Biosensors

### 2.1. Biosensors Operating in Tears

The complex aqueous fluid secreted by lachrymal glands, i.e., human lachrymal liquid or tears, apart from water and electrolytes, carry low molecular weight organic compounds, proteins, enzymes, as well as other biomolecules. Owing to the physiology of the eye, different lachrymal secretions can occur, and three kinds of tear, basal, reflex, and psycho-emotional tears [26], each substantially differing in composition, can be observed. Basal tears are produced in small quantities, 0.5–2.2 µL min^−1^ [27], to maintain a film on the corneal surface, ensuring corneal homeostasis and visual integrity. Reflex tears result from increased lacrimation, 7–23 µL min^−1^ [28], in response to damage to the ocular surface by foreign bodies, including contact lenses [29], chemicals, wounds, and inflammation [26]. Psycho-emotional tears are provoked by cerebral stimuli of psychogenic origin. While basal tears result from spontaneous neuroglandular activity and reflex tears are the result of external sensorial stimulation, and both kind of tears are expletively purposeful, psycho-emotional tears are triggered by cognitive and emotional brain processes, and are of no apparent use for the eye [26].

Several compounds found in tears have diagnostic potential, e.g., glucose, lactate, ascorbate, and neurotransmitters, e.g., dopamine and norepinephrine [30]. Hence, many diseases and ailments can be diagnosed by analyzing the composition of tear fluid, and an adequate summary of this can be found in a recent review [31]. Illustrating the potential of tear-based bioanalyte sensing, glaucoma patients suffer from lower than average tear neurotransmitter concentrations, and determination of catecholamines in tears has been advocated in glaucoma diagnosis [30]. Also, the baseline concentration of the stress marker norepinephrine is high enough for detection, and since the concentration of this is likely to increase during psychological and physical challenges, it should be possible to realize a non-invasive tear-based stress sensor.

Tear sampling is expressively non-trivial and the established methods all suffer from shortcomings. For example, tears can be absorbed using Schirmer strips resting on the lower eyelid, but the procedure tends to collect cellular as well as secreted proteins, and the physical presence of the strip can cause mechanical stimulation of the corneal and conjunctival epithelium, provoking release of reflex tears [32,33]. Consequently, the composition of the samples thus collected most likely differs from that in native basal secretion [34]. If, instead, microcapillary tubes are used to draw tears from the reservoir within the conjunctival sac, while seemingly less invasive than Schirmer strips, collecting basal tears with this method can be tedious and time consuming, and to accumulate volumes adequate for analysis it may be necessary to pool samples [32,35]. In addition, the microcapillary tube method shares the Schirmer strip reflex tear issues. To conclude, the small volumes collected along with low concentrations of tear bioanalytes could explain the large discrepancies in concentration values reported, and it appears that authentic and accurate bioanalyte concentrations in human tears are yet to be ascertained.

In order to bypass tear sampling issues, several research teams have turned their attention to contact lens-based biosensors. As far as can be asserted, the first attempt to design an on-lens electrochemical biosensor coincided with efforts to realize electronically augmented contact lenses for bionic eyesight [36,37]. The lens assembly incorporated a 100 μm thick poly(ethylene terephthalate) (PET) film, fitted with an amperometric sensor, in which the Pt working electrode was connected to an indium-tin oxide (ITO) substrate carrying a self-assembled monolayer of glucose oxidase (GOx). Potential control was achieved using an external Ag|AgCl reference electrode [36,38]. Glucose amounts were indirectly assessed, based on electrochemical oxidation of H_2_O_2_ (Reaction (1)), formed as a co-product in the GOx catalysed oxidation of glucose (Reaction (2)).

(1) $$H_{2}O_{2}\overset{Pt}{\to }2H^{+}+O_{2}+2e^{-}$$

(2) $$D\text{-}glucose\overset{GOx}{\to }D\text{-}gluconolactone+H_{2}O_{2}$$

**Figure 1:**
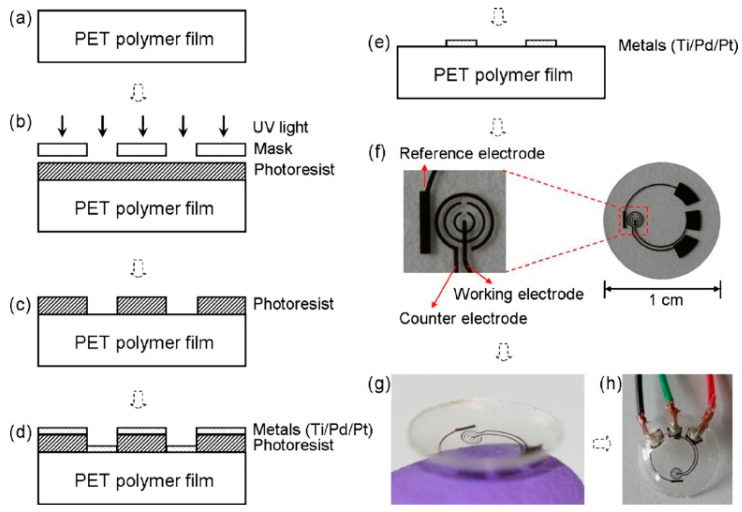
The sensor fabrication process and results: (**a**) a clean PET substrate is prepared; (**b**) the substrate is covered with a photoresist and exposed to UV light through a mask; (**c**) the photoresist is developed; (**d**) thin metal films are evaporated on the sample; (**e**) after lift-off, the metal pattern remains on the surface. After this step, the sensor is cut out of the polymer substrate and heat molded to the contact lens shape and functionalized with enzymes; (**f**) images of a sensor after it has been cut out of the substrate; (**g**) image of a completed sensor after molding held on a finger; (**h**) the sensor hardwired for testing. Reproduced from [5] with permission from Elsevier.

The sensitivity of the first/original sensor was unsatisfactory, and an improved biosensor design was called for [5]. Thus, the second generation sensor was essentially a PET film fitted with a three electrode system with an integrated reference electrode (Figure 1), distinguishing the novel sensor from the original [36]. Bio-modification relied on a GOx/titania sol-gel membrane and the sensor was provided with a Nafion^®^ layer to decrease the influence of other redox active species, like ascorbate, lactate, and urea, present in the lachrymal fluid.

At a constant voltage of 0.4 V, the amperometric sensor showed quite promising performance in glucose containing solutions, and excellent current and glucose concentration linearity in a physiologically relevant glucose concentration range. However, the influence of interfering redox species shifted the detection limit by about one order of magnitude. After storage for two and four days in buffer at 4 °C, the residual current response was 80% and 55%, respectively, of the initial current. In order to minimize the contribution from interfering substances additional control working and counter electrodes were fitted, resulting in a so called dual sensor setup [39]. Control electrodes were assembled using the same approach as for the signal electrodes, but the bio-modification with a GOx/titania sol–gel membrane and electrode coating with Nafion^®^ was omitted. Background currents obtained from the dummy sensor were subtracted from the bioelectrocatalytic sensing electrode currents, and measurements using a physiologically accurate flowing eye model confirmed that the dual sensor did indeed lower the glucose detection limit, but it was still too high for real practical applications. In the on-lens version of the biosensor, wirelessly powered and with an integrated telecommunication circuit, the control electrodes were covered with deactivated GOx to subtract background currents more accurately [40,41]. The sensitivity of the on-lens biosensor, tested on a model human eye, was found to be 18 μA cm^−2^ mM^−1^ with a linear response in the 0–2 mM glucose range in a tear mimicking buffer solution. The artificial tear solution contained redox active species, i.e., ascorbate, lactate, and urea, at concentrations typical for human tears, and representative tear proteins, i.e., lysozyme, mucin, and albumin, all of which may affect the biosensor performance. The residual bioelectrocatalytic current was 97.4% of the initial after 12 h of storage in buffer at 4 °C, and 84.3%, 67.2%, and 54.2% after one, two, and four days, respectively, without any loss of current vs. glucose concentration linearity. The on-lens sensing platform was powered by an on-chip 1.2 V supply, consuming just 3 µW, and could also be wirelessly powered from a distance of 15 cm [40]. Google and Novartis foreshadowed that commercially available glucose monitoring contact lenses could be expected already in 2015 [42]. However, the companies did not deliver on their promise, and to the best of our knowledge, the project is completely terminated. One of the possible reasons for that might be attributed to the partial discharge of reflex tears in response to damage to the ocular surface. Reflex tears have different composition compared to basal lachrymal liquid, and even for one individual, bioanalyte concentrations could vary from one day to another. To mitigate or bypass the problems related to contact lens based biosensors, usage of soft, highly flexible, air breathing materials is suggested.

**Figure 2:**
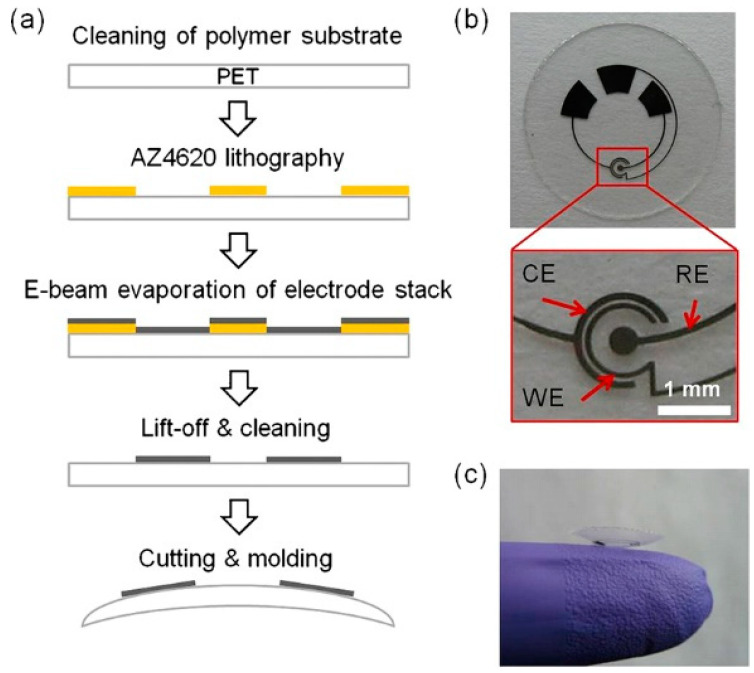
Lactate sensor on a contact lens. (**a**) Schematic representation of the assembly process for sensors on a transparent PET substrate which is molded to a contact lens. (**b**) Flat substrate with sensing structure, interconnects and electrode pads for connection to the external potentiostat; WE—working electrode, CE—counter electrode, RE—reference electrode. (**c**) A completed contact lens sensor held on a finger. Reproduced from [43] with permission from Elsevier.

In addition to glucose detection, the sensor design was adapted to contact lenses with integrated lactate biosensors (Figure 2) [43].

Lactate was detected based on oxidation of lactate to pyruvate by molecular oxygen, catalyzed by lactate oxidase (LOx) (Reaction (3)), with subsequent electrochemical oxidation of H_2_O_2_ at a Pt working electrode (see Reaction (1)).

(3) $$L\text{-}lactate+O_{2}\overset{LOx}{\to }pyruvate+H_{2}O_{2}$$

LOx and bovine serum albumin were co-immobilized on the sensing area, and the proteins were simultaneously crosslinked using glutaraldehyde, and to prevent enzyme leakage and to reduce the influence of interfering redox species the electrodes were sequentially covered with polyurethane and Nafion^®^. In PBS, the sensitivity reported was 53 μA cm^−2^ mM^−1^, with a response time of 35 s, and a linear detection range of 0–1 mM lactate. An interfering signal owing to direct oxidation of ascorbic acid on the Pt surface was successfully eliminated by using a dual sensor setup (vide supra). The sensor maintained full functionality after 24 h of storage in buffer at room temperature.

Mitsubayashi et al. managed the design of an on-lens glucose biosensor differently [44,45]. Drawing on a two-electrode setup, i.e., a Pt working electrode and a combined Ag|AgCl reference/counter electrode, the amperometric biosensor was affixed to a 70 µm thick polydimethyl siloxane (PDMS) membrane, attached to a soft contact lens made of the same material. GOx was immobilized from a co-polymer of 2-methacryloyloxyethyl phosphorylcholine and 2-ethylhexylmethacrylate (PMEH) mixture, and the GOx-polymer layer was additionally covered with a PMEH membrane to prevent enzyme leakage. Glucose sensing was based on electrochemical oxidation of H_2_O_2_ on the Pt electrode, vide supra, Reactions (1) and (2), and a linear relation between current output and glucose amount was obtained in buffer solutions with 0.03–5 mM glucose. Testing the sensor in wearable mode using rabbit models gave excellent results and it was also demonstrated that the glucose concentration in tears traces the changes in blood glucose amounts with a delay of approximately 10 min. The sensitivity of biosensors could be substantially improved by building 3D micro-pillar electrodes, which have up to three times higher surface area when compared to the flat analogues [46].

**Figure 3:**
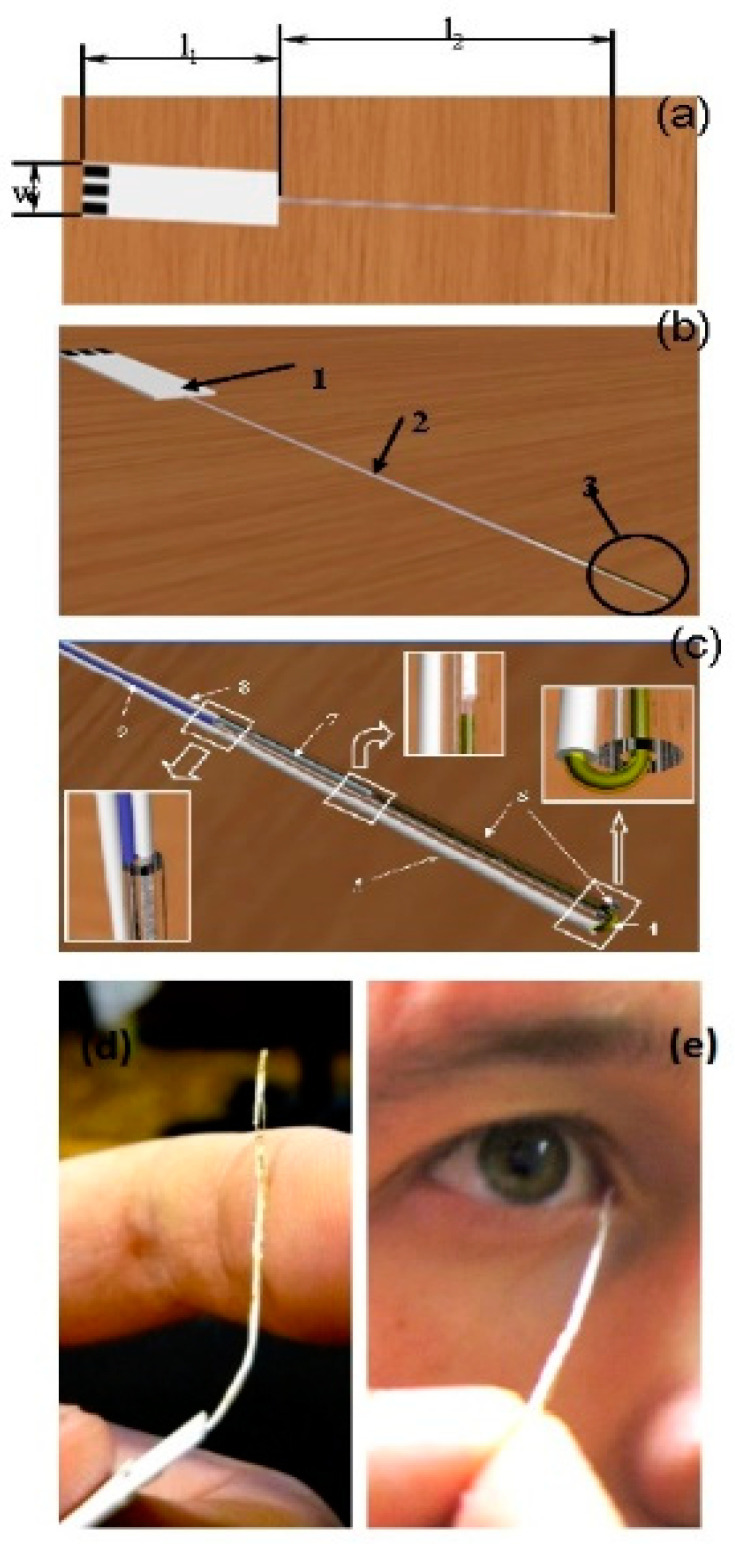
Schematic view of the flexible non-invasive micro-biosensor. (**a**) l1—length of a handle (from 10 mm up to 50 mm, optimum 20–30 mm), l2—length of a flexible sampling part (from 40 mm up to 100 mm, optimum is 50–60 mm), w—width of the handle (from 5 mm up to 10 mm). (**b**) 1—the handle with three electrical contacts, 2—the flexible sampling part (total diameter from 0.05 mm up to 0.5 mm), 3—the flexible part of the device with a microcell. (**c**) Flexible part of the sapling device including a microcell. 4—working electrode (diameter from 0.01 mm up to 0.2), 5—insulated part of the working electrode, 6—polymeric tube (internal diameter from 0.015 mm up to 0.25), external diameter from 0.02 mm up to 0.4, length from 1 up to 40 mm) fused to the insulated part of the working electrode, 7—counter and reference electrode (diameter from 0.01 mm up to 0.2 mm), 8—insulated part of counter and reference electrode, 9—checking/control electrode (diameter from 0.01 up to 0.2 mm). Distance between ends of working and opposite electrodes from 0.01 mm up to 1 mm. (**d**) Photograph demonstrating the flexibility of the biodevice. (**e**) Photograph of authentic sampling. Reproduced from [47] with permission from Springer.

Some non-invasive tear-based biosensors cannot easily be repurposed as wearable devices. A flexible electrochemical microbiosensor has been designed for quantitative analysis of glucose, ascorbate, and dopamine in nanovolumes of human lachrymal fluid (Figure 3) [47]. The biodevice is based on glucose dehydrogenase (GDh), rather than GOx, and ascorbate and dopamine are analyzed electrochemically without pre-enzymatic reactions. GDh was immobilized on a gold microwire modified with carbon nanotubes and an osmium redox polymer. A capillary microcell, with a working volume of 60–100 nL and a sampling deviation of about 7%, was constructed for tear sampling. To check if the microcell was properly filled with buffer or a tear sample, a control electrode was introduced into the construction (Figure 3c). The electrode was used to measure the electrical resistance of a fully filled nanovolume cell. The mechanical flexibility is one of the most important features of the prototype and it allows direct collection of tears with minimal risk of damage to the eye (Figure 3d,e). Based on the experimental results, the authors concluded that the flexible and non-invasive prototype could be converted into a user-friendly microbiosensor, suitable for detection of blood bioanalytes, including glucose, in human lachrymal fluid [47].

### 2.2. Biosensors Operating in Saliva

Saliva is an oral fluid that is mainly produced by three pairs of major salivary glands: parotid (inside of the cheeks), sublingual (under the tongue), submandibular (bottom of the mouth), and a large number of minor salivary glands [48,49,50,51]. Moreover, saliva is a clear, viscid, complex [52,53,54,55,56], colourless, odourless fluid, with a pH in the 6.6–7.1 range [50,57]. Saliva is a watery substance (99.5% water [58]) that incorporates different elements like bacteria, leukocytes, epithelial cells, crevicular fluid [53,58], hormones, ions [54,56], enzymes, proteins, nucleic acids, antimicrobial constituents, cytokines, and antibodies [56,57]. These different components, originally from the blood, can diffuse through para-cellular or trans-cellular pathways in the oral cavity, adding to the complexity of saliva [49,55]. Additionally, the oral cavity also comprises a large number of bacteria (oral microbiome) [49].

Saliva analysis has a considerable potential regarding general health status monitoring [48,59]. Saliva carries a broad range of biomarkers [49] that can be used for clinical analysis and diagnostic testing of various diseases [49,50,54,56,60]. Moreover, because many biomarkers found in saliva are passed directly from the bloodstream, changes in saliva composition indicate the current health status of the examined person [56,60]. The correlation between the blood and saliva concentration of different biomarkers and metabolites like lactate [48,53,60,61], ethanol [48,53], cholesterol [53,60,61], or glucose [49,54,60,61,62,63,64] has been established. Therefore, saliva analysis gives the opportunity to monitor and surveil the emotional, hormonal, nutritional, and metabolic state of the human body [54,55]. Additionally, utilizing saliva as a diagnostic fluid offers various advantages, e.g., a painless and non-invasive method for diagnostics and monitoring [48,49,52,53,54,55,65,66] relying on a simple and fast collection method [48,49,55,67,68]. Moreover, sample collection is conveniently trivial [48,49,55], not privacy invading for the patient [49], and does not require special laboratory equipment or trained medical personnel [49,55,60,65,66]. Additional to that, saliva can be used as an alternative fluid because it is generally safer and has a lower contamination risk compared to blood tests [48,49,54,66]. Therefore, saliva offers the possibility to analyze various biomarkers in an easy accessible, reliable, cost-effective way [48,49,56,65,67] and can realize a multiplex detection of metabolites with high sensitivity and selectivity [61]. On the top, using saliva can realize dynamic measurements of biochemical markers with an read out in real time [54], or a portable biosensing platform for health care monitoring [54,69].

These benefits and the different prospective options led to an increased focus in this research field with the aim of assay developments and technological advancements for the detection of various salivary biomarkers to improve clinical diagnosis, management, and treatment [48]. Normally, detection of salivary biomarkers is performed by using laboratory-based assay methods that involve multiple steps in a time-consuming process. This includes, e.g., collection/transfer of the sample followed by processing in the laboratory [48,65]. Therefore, the demand for fast, simple, inexpensive, reliable, accurate, portable, and on site (point-of-care) quantification of salivary biomarkers through the use of biosensing technology increased and is supported by the progress in nanotechnology [48,60,65].

Various biomarkers or molecules can be detected in saliva, e.g., the cytokines interleukin-6 [70,71], interleukin-8 mRNA [72,73,74], interleukin-8 protein [72,73,74], cancer antigens [48] like carcinoembryonic antigen (CEA) [75,76], cancer antigen 125 (CA125) [76] and Her-2/Neu (C-erbB-2) [76,77], VEGF165 [78], TNFα [79], or cytokeratin-19 antigen (Cyfra 21-1) [80]. Moreover, lactate [48,54,58,81,82,83,84], glucose [48,60,62,85,86,87,88,89,90,91,92,93,94,95,96], or hormones [48,66], like cortisol [48,49,51,65,66,97,98,99,100,101,102], and cortisone [99,103,104] are of interest to detect as well. Furthermore, molecules like uric acid [49,54,105], urea [106], L-tryptophan [107] or different enzymes, like α-amylase [48,66,108,109,110] or aspartic peptidases [111,112] are found in saliva. Additional to that, the detection of bacteria, viruses, and whole cells is possible, like *Helicobacter pylori*, *Streptococcus sanguinis*, *Escherichia coli*, *Staphylococcus aureus*, *Chikungunya viruses* [113,114,115,116,117,118] or different antibodies [48,66,114,119], e.g., HIV [48,66,120]. On the top, different drugs [49,51,80,121], like alcohol [53,66] and cocaine [66,122], as well as neurotransmitters [49,66,123] can be determined in saliva. The related diseases to the different biomolecules are cancer [51,70,73,74,75,76,77,79,80,124], oral cancer [56,67,72,108,109], breast cancer [56,67], diabetes [49,60,67,85], and different cardiovascular diseases [68,108,109]. Moreover, diseases of the oral cavity like periodontal infections [56,67,125] or caries risk assessments [56] can be done. Additional to that, infections caused by bacteria or virus-like viral hepatitis A,B,C [51,67], related to infections, or cardiovascular diseases [68,108,109,118] or metabolites, like lactate that can diagnose severe sepsis, septic shock, and many more [83,126].

**Figure 4:**
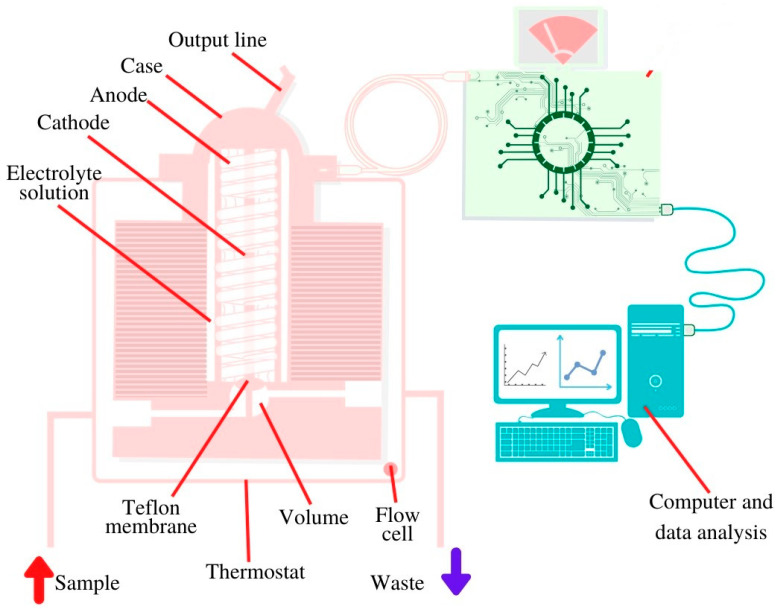
Schematic view of the salivary glucose biosensor inside installed in a 37 °C thermostat.

The correlation between the concentration of glucose in blood and in saliva was established by different groups [49,54,60,61,62,63,64] and, hence, the opportunity was given to realize a biosensing platform for the detection of salivary glucose [48,60,62,85,86,87,88,89,90,91,92,93,94,95,96]. For the realization of an enzyme-based glucose sensitive sensor, a screen-printed sensor chip was produced by a layer-by-layer assembly process to functionalize the working electrode surface. The multilayer films were composed of single-walled carbon nanotubes functionalized with carboxylic groups and three repeated layers of chitosan/gold nanoparticles/GOx to achieve the best glucose-sensing performance using amperometry [62]. To enable constant monitoring over a specific period of time, an oral biosensor was imbedded in a mouthguard and miniaturized to a detachable ‘cavitas sensor’ based on an enzyme membrane with GOx (Poly (MPC-co-EHMA)). The device was integrated with a wireless transmitter, based on a platinum and silver/silver chloride electrode, with amperometric read out, and real time measurement of the glucose concentration was achieved [88]. Another group used a bimetallic, bifunctional electrode where a platinum surface was patterned with nanostructured gold fingers with different film thicknesses. The gold fingers were functionalized with GOx by using selective adsorption of a self-assembled monolayer onto gold fingers. GOx on gold acted on glucose and the hydrogen peroxide formed was detected on the platinum sites. The read out was based on cyclic voltammetry and impedance spectroscopy [96]. Another glucose sensor was a disposable saliva nano-biosensor. The platinum working electrode was functionalized with single-walled carbon nanotubes and multilayers of chitosan, gold nanoparticles, and GOx, generated by using a layer-by-layer assembly technique and the final read out relied on amperometry [60]. Last but not least, the detection of salivary glucose can be achieved based on flow injection analysis combined with an amperometric O2 electrode [127]. Glucose sensor presented in Figure 4 consists of transducer constructed of silver anode and golden cathode covered with potassium chloride. The electrode is also covered with Teflon membrane with immobilized GOx, a test solution is injected using a micro syringe [127].

The correlation between the concentration of cortisol in saliva and blood has been established, providing the opportunity to use the detection of this for the development of various biosensors [98,128,129]. A highly sensitive and non-invasive electrochemical immunosensor for salivary cortisol sensing was developed by using an NiO thin film-based label-free electrochemical immunosensor. For this purpose, cortisol antibodies were immobilized with EDC and NHS on the electrode surface and detected with differential pulse voltammetry [100]. Another electrochemical label-free immunosensor was established with interdigitated microelectrodes and anti-cortisol antibodies, which were covalently immobilized on self-assembled monolayers of dithiobis(succinimidylpropionte) (DTSP). The different concentrations of cortisol were evaluated using cyclic voltammetry [101]. Another electrochemical immunosensor was based on one-dimensional ZnO nanorods and two-dimensional ZnO nanoflakes that were synthesized on gold-coated substrates, followed by immobilizing anti-cortisol antibodies; the detection of cortisol was performed by cyclic voltammetry [102].

For the detection of TNF-α in human saliva and serum samples, a disposable immunosensor based on thin indium tin oxide films, covered by a new semi-conductive conjugated polymer, was developed. The read out was realized by electrochemical impedance spectroscopy [79]. Another electrochemical biosensor platform for TNF-α cytokines detection in both artificial and human saliva was introduced. This fully integrated platform was developed to detect varying cytokine biomarkers by using eight gold working microelectrodes. TNF-α antibodies (anti-TNF-α) were immobilized on gold working electrodes through functionalization with carboxyl diazonium; detection relied on amperometry [59].

Another group developed a graphene-based fully integrated portable nanosensing system for the detection of interleukin-6. This new sensor was based on the permittivity of a HfO_2_ dielectric layer in the buried-gate graphene field effect transistor. Moreover, the detection was performed by the immobilization of aptamers and the data transfer was realized by a wireless connection via WiFi [70]. An additional possibility to detect interleukin-6 was based on a magneto immunosensor design. Here, the anti-IL-6 antibodies were immobilized on carboxyl-functionalized magnetic microparticles. This immunoassay created the signal amplification by using poly-HRP-streptavidin conjugates that were immobilized on screen-printed carbon electrodes. The final detection was realized with amperometry [71].

Two biosensors were developed for the simultaneous detection of interleukin-8-protein and interleukin-8-mRNA. One of the sensors was an amperometric magnetosensor which allowed the direct determination of both biomarkers with an antibody and a hairpin DNA. The specific hairpin DNA probe and the antibody were coupled on screen-printed carbon electrodes and used the HQ/HRP/H_2_O_2_ system [72]. The second biosensor was also based on amperometric detection but used biotin and fluorescein dual-labeled hairpin probe for IL-8 mRNA and biotinylated human IL-8 monoclonal antibody on a gold integrated electrode array. Moreover, the group used a conducting polymer as a supporting film to improve sensor performance [73].

The determination of lactate was accomplished with an electrochemical enzyme probe (LOx), by using a dual electrode system (two platinum electrodes), and an enzyme membrane that was imbedded between a cellulose acetate membrane and a polycarbonate membrane. The changes in the lactate concentration were measured with the relating current response, after injecting the salvia sample with PBS and by subtracting the inactive electrode response from the active electrode response [81]. Another approach for detection lactate was to use a cavitas sensor (placed in the oral cavity). This wearable biosensor on a mouthguard is based on an Ag/AgCl reference electrode and contacts (for interfacing the electrochemical analyser) on a flexible PET substrate. Moreover, it was based on the integration of a printable enzymatic electrode, with LOx, on a mouthguard, and detection of the hydrogen peroxide formed. The mouthguard sensor was made of a Prussian-Blue transducer and a poly-orthophenylenediamine (PPD)/LOx layer. The amperometric measurements were realized with the connected PB-PPD-LOx system [82].

The detection of uric acid was realized by a sensor that consisted of an uricase-modified screen-printed electrode system and was also integrated in a mouthguard platform. The whole sensor was based on a flexible PET substrate, including a Prussian-blue transducer and immobilized uricase enzyme. This wearable sensor was connected to a wireless device via Bluetooth for data collection and allowed real-time and continuous measurement of the uric acid concentration with an amperometric read out [105].

Additionally, the detection of orexin A (a neurotransmitter), was accomplished by using a gold field effect transistor-based biosensor that was modified with zinc oxide. For this purpose, a novel peptide recognition element was synthesized and coupled to the electrode surface [123].

For the detection of the protein Pf HRP2 (*Plasmodium falciparum* histidine-rich protein 2) an enzyme-free electrochemical immunosensor was developed. This sensor was based on an immunosandwich format and used a competitive detection principle with methylene blue, hydrazine, and platinum nanoparticles. For this purpose, the specific antibodies were labelled with methylene blue and immobilized on an indium tin oxide electrode. The read out was realized with chronocoulometric measurements [130].

Furthermore, the sensing of tryptophan, a standard amino acid, was reported by a group using two screen printed electrodes modified with multiwall carbon nanotubes on gold electrodes. Additionally, the electrode surface was modified with aptamer molecules to determine tryptophan, followed by impedimetric read out [107]. Another approach was made, by using magnetic multiwalled carbon nanotubes as nanocarrier tags for the detection of human fetuin A with impedimetric read out. Moreover, the electrochemical immunosensor was realized by using the linker molecule diazonium, followed by the immobilization an anti-human fetuin A-antibody and coupled HRP [131].

For the detection of cocaine, a solid-state probe based on an electrochemical aptasensor was developed. The sensor principle was based on a layer-by-layer self-assembled multilayer with ferrocene-appended poly(ethyleneimine) on an indium tin oxide electrode array. Additionally, gold nanoparticles were coupled to the electrode surface. The final read out was realized by using differential pulse voltammetry and measuring the related signal of ferrocene [122].

The development of a biosensor can be also used to evaluate the efficiency of a treatment in the field of pharmacology. For this purpose, another electrochemical aptamer-based sensor was introduced to detect ampicillin. The aptamers were immobilized on a gold electrode, followed by a blocking step with MCH to avoid unspecific binding. When ampicillin is bound by the methylene blue modified aptamer, the aptamer conformation changes; the measurement of the change was realized with the methods of alternating current voltammetry and square wave voltammetry [80].

One group developed a biosensor for the detection of *S. pyogenes*. This sensing platform was based on the immobilization of antibodies on a gold surface. They used screen printed gold electrodes to create a polytyramine (Ptyr)-based immunosensor. Accordingly, NeutrAvidin was coupled to the Ptyr amine group, followed by the immobilization of biotin tagged antibodies against *S. pyogenes* [113].

For the detection of HIV antibodies, an electrochemical peptide-based sensor was introduced. This sensor used the incorporation of extra amino acids that acted as a target recognition element and antifouling agent on gold electrodes. Moreover, the peptide probe was thiolated (coupling to the electrode surface) and methylene blue-modified (detection). With the binding of the HIV antibody, the methylene blue related current decreases and allowed a read out with alternating current voltammetry and cyclic voltammetry [114].

Despite many achievements, a better understanding is needed for the relation between biomarker concentration in blood and in saliva to improve the reliability of various diagnostic platforms, to provide accurate oral monitoring applications, and to develop highly sensitive sensors [54]. In particular, higher sensitivity is an important requirement due to the fact that the concentration of many important biomarkers in saliva are lower than in blood, e.g., proteins with ∼30% lower concentration in saliva [54,68]. Additional to that, metabolite measurement in saliva is complicated by the presence of bacteria, epithelial cells and leukocytes [53]. Moreover, when sampling saliva, challenges still remain, e.g., including sampling standardization since the concentration of different compounds in saliva depends on the flow rate and the flow rate varies in response to any pre-sampling stimulation [53]. Additionally, saliva viscosity can vary substantially, which makes it more difficult to provide a reliable testing platform [68]. Furthermore, after the saliva collection, the sample must be put on ice to reduce growing of microorganisms [53]. Moreover, various sensing problems can occur after/before/during food and drink consumption because it can interfere with the analyte sensing [52,54,68]. Furthermore, future development should focus on anti-fouling strategies. These strategies are needed because a high concentration of proteins in saliva, e.g., mucins and proteolytic enzymes, will cause nonspecific adsorption on the electrode surface; the oral microbiome will produce a biofilm, and consequently, lower the life time of sensors in general [52,54,69]. This poses serious challenges, especially for real-time measurements and long-term monitoring of biomarkers since saliva is not only a complex solution but also a dynamically changing one [52,54,68].

Moreover, for development of devices and sensing platforms, different requirements should be met, e.g., on-body/in-vivo compatibility (compatible biomaterials for device and system), including an effective device encapsulation (electronic interface, power supply, wireless communication) and will alleviate the toxicity of the whole sensor [22]. Furthermore, for an in vivo sensing platform the sensor should be mechanically robust and securely fixed in the oral cavity and fit with the mouth anatomy and spatial ranges [55]. This increases the comfort for the wearer and a firmly fixed position will not change with mouth muscle movements [52].

Multiplex sensing platforms have also been introduced. One sensor was developed for the detection of glucose, lactate, and cholesterol, which was based on an organic electrochemical transistor (OECT) microarray integrated with a pumpless “finger-powered” PDMS-based microfluidic system. The group combined a biofunctionalization method and electrically isolating layer between the devices to decrease the background interference and crosstalk for improving the sensing abilities. Additionally, they immobilized GOx, LOx, cholesterol oxidase, and bovine serum albumin (control) on each electrode separately and realized the read out by using chronoamperomerty and the channel current response [61].

Another group developed a flexible organic electrochemical transistor to detect uric acid and glucose. For this purpose, the gate electrodes of the transistor were modified with positively/negatively charged bilayer polymer films and enzymes (uricase and GOx). Additionally, the platinum electrode surface was modified with graphene flakes and Nafion, followed by PANI (polyaniline, a conducting polymer). The whole sensor was based on the selectivity for H_2_O_2_ and measured the resulting channel current response of the transistor [132].

For future developments, power supply and communication challenges should be addressed. This comprises for instance, to find and implement new solutions/sources for the power that is needed for sensing/detection of biomarkers, data processing/data collection, data transfer/communication. Alternative strategies could include incorporation of batteries, biofuel cells, solar cells, or thermoelectric generation of power [54]. Moreover, communication challenges include the overall integrity of wireless communication and a long-distance data transmission between the sensor and the device for the connected read out. Additionally, data security problems will require a safer collection and storage of the biomedical data because the sensor is monitoring the patients’ health status in real time, remotely, and continuously [54]. All in all, considerable validation studies would be necessary to establish sensors in clinical applications and to make is accessible for the general public [54].

### 2.3. Biosensors Operating in Sweat

Other sensing technologies for measurement of various analytes utilizes the biofluid sweat [23,133]. Sweat contains several compounds that provide helpful medical information about health and metabolic status, physiological state, and disease states [23,24,58,134,135,136,137]. The outer skin surface comprises a high number of sweat glands that are densely and widely distributed all over the body [54,138]. These sweat glands produce and excrete an acidic fluid directly to the outer skin surface through microscale pores [23,68]. Moreover, sweat includes various molecules, e.g., lactate [133,138,139,140], glucose [69,134,137,141,142], cortisol [23,54,135,142,143,144], testosterone [23], uric acid, as well as larger molecules, like proteins, peptides, and cytokines [24,54,142],

Additionally, the detection of analytes in sweat is feasible due to the given correlation between the blood analyte concentration and the excreted sweat analyte concentration [138,145,146]. For analytes like hormones (cortisol, testosterone), potassium, and different drugs, e.g., alcohol, a strong sweat-blood correlation was proven [23]. Moreover, an understanding has been established about the glucose-level relation between sweat and blood and its potential use in diabetes monitoring [23]. The correlation between sweat and blood concentration for lactate or urea is not so far approved, but lactate/urea measurements in sweat can give an indication of the health status of the examined subject [23].

Therefore, utilizing sweat as a diagnostic biofluid offers the possibility of non-invasive diagnostic platforms [134,135,136,137,138,147] with effortless sample collection [23,53,54], compared to blood [24]. Moreover, sweat biosensors are readily adapted to wearable sensing system [54,133,138] for real-time, dynamic, and continuous measurement of biomarkers [23,54,133]. Over recent years, given the progress in nanotechnology, development of novel sweat-based biosensors has grown [23,24].

When collecting data from sweat, the sensor must be in close contact with the skin, ideally with a planar fit. Moreover, sweat biosensors are mostly produced with a flexible substrate for contact with the skin surface, enabling the option of a wearable sensor platform. Both characteristics, i.e., planar fit and flexibility, ensure proper sweat sample collection and a lower required sample volume [23,24,148]. Additional requirements for a wearable sweat sensor are the following: a fast response time of the detected analyte, high stability, selectivity, and sensitivity under environmental conditions [24]. A fast response time of the sensor can be realized and accomplished by using e.g., electrochemical read out methods, such as amperometry and electrochemical impedance spectroscopy [24]. The integration of electrochemical methods for the sensor development of sweat biosensors has different advantages in terms of low cost, high performance, and device portability [136]. However, a significant drawback when performing quantitative assessments is normalization of the sampled volume. One way to mitigate this is by also incorporating monitoring the sweat flow rate, e.g., by measuring the change in sweat generation rate by skin impedance. However, without a detailed fluidic model between the sweat glands and sensors, the sweat rate does not predict actual biomarker sampling intervals [149].

Enzymes, which are immobilized on the surface of the working electrode via covalent cross linking or bonding [150], are widely used as biorecognition elements in sweat-based biosensors. Some examples include GOx [24,137,141,151,152], LOx [137,139,151,153,154], alcohol oxidase (AOx) [140,155,156,157,158,159]. For an improved enzyme immobilization and at the same time a maximized surface concentration and an increased surface area, nanoparticles with silver [91,160], gold [161], and nickel [162], as well as nanofibers, such as zinc oxide [23], can be used. This modification step results in higher electrode response, faster sensor response time, and a higher selectivity to the investigated analyte [23].

**Figure 5:**
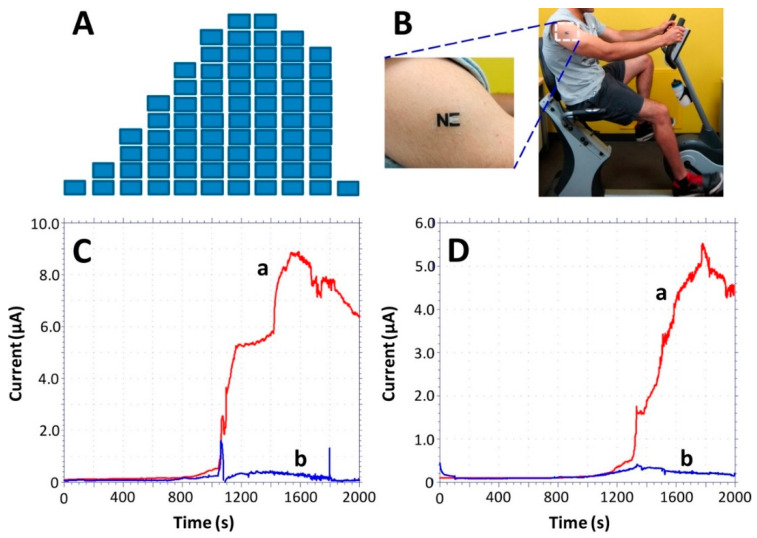
Monitoring of sweat lactate during 33 min of cycling exercise while adjusting the work intensity. (**A**) Exercise resistance profile on a stationary cycle. Subjects were asked to maintain a constant cycling rate while the resistance was increased every 3 min for a total evaluation of 30 min. A 3-min cool down period followed the exercise. (**B**) An “NE” lactate biosensor applied to a male volunteer’s deltoid; (**C**,**D**) Response of the LOx- (a) and enzyme-free (b) tattoo biosensors during the exercise regimen (shown in part A) using two representative subjects. Constant potential, 0.05 V (vs. Ag|AgCl); measurement intervals, 1 s. Reproduced from [140] with permission from the American Chemical Society.

For enzymatic sensing of metabolites, like glucose, lactate, ethanol, and uric acid, an amperometric read out is often used [148,153,157]. The detection of lactate is realized with the immobilization of the enzyme LOx and was demonstrated by different working groups [138,139,140,154]. Already, in the early nineties, an amperometric H_2_O_2_ based biosensor was developed to detect lactate and was based on enzyme immobilized between a polycarbonate membrane and a polytetrafluoroethylene (PTFE) blocking membrane [139]. Furthermore, a flexible array patch with LOx and a Prussian blue/gold electrode [137], a temporary tattoo using carbon ink (Figure 5) [140] was introduced to monitor diseases, like pressure ischaemia, peripheral arterial occlusive disease, or hypoxia [137,140,151]. Another accomplishment drew on two electrocardiogram electrodes combined with the detection of lactate (three electrode system). The so called “Chem-phys”-patch is based on a biocatalytic layer with LOx and modified Prussian blue on a polyester sheet [154].

The possible sensing of the metabolite glucose in sweat was demonstrated by different working groups for the detection of diabetes mellitus [24,137,141,151,152]. This included, e.g., a flexible array patch with immobilized GOx on a Prussian blue/Au electrode [137], multi-analyte glasses based on GOx attached to a gold electrode [151], or a graphene based stretchable patch using GOx on a Prussian blue/graphene-Au electrode [152]. For another sensing platform gold and platinum alloy nanoparticles were electrochemically deposited on a reduced graphene oxide surface and chitosan-GOx composites were integrated onto the modified surface [141].

Moreover, the detection of another metabolite, ethanol, was successfully accomplished, using a skin surface-based sensing device for determining the blood’s ethanol content by monitoring transdermal alcohol concentration. For this purpose, two enzymes were used, GOx/horseradish peroxidase (GOx/HRP), immobilized on a graphite-Teflon electrode [158]. Other developments were a temporary tattoo with AOx immobilized either on Prussian blue [157] or a wearable patch using platinum electrodes [159].

**Figure 6:**
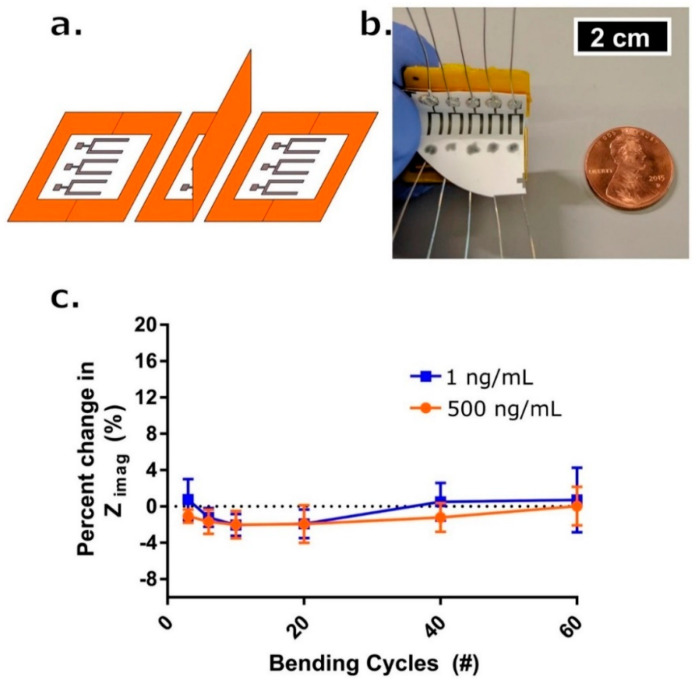
(**a**) Schematic drawing for one complete bending cycle of the sensor. The cycle is comprised of (left) unbent state, (middle) 90° flexion motion bend, and (right) return to unbent state at which point a measurement occurs. (**b**) Picture of bending apparatus with an affixed sensor array affixed. Penny for reference. (**c**) Percent change in Zimag impedance with respect to the initial measurement post-cortisol dosing and 7-min incubation time (blue box—1 ng/mL, red circle—500 ng/mL) after # of bending cycles (*n* = 3). Error bars are standard error of the mean. Reproduced from [144] with permission from Springer Nature Limited.

Impedance-based sensors were developed to detect different metabolites in sweat, e.g., glucose [135,143], lactate [163], or biomarkers, like Interleukin-6 [142] or cortisol [135,142,144]. For the detection of cortisol, MoS2 nanosheets were functionalized with cortisol antibodies to create a non-faradaic label-free cortisol biosensor (Figure 6) [144]. One working group developed flexible, wearable, nanoporous tunable electrical double layer biosensors with a bio-functionalized area of Zinc oxide (active region) to detect cortisol in sweat due the changes of impedance caused by the modulation of the double layer capacitance [135]. The same working group introduced a lancet-free and label-free diagnostic platform to detect glucose and cortisol in sweat. They used again zinc oxide based flexible bioelectronics of stacked metal/metal-oxide (gold/zinc oxide) thin films within porous polyamide substrates. Additionally, antibodies specific to GOx and for cortisol were attached to the zinc oxide region [143]. Moreover, this group also developed a sensor which enhanced the stability of biomolecules by using room temperature ionic liquids. In this paper they used sensors on nanoporous, flexible polymer membranes functionalized with antibodies to detect interleukin-6 (IL-6) and cortisol in human sweat [142].

**Figure 7:**
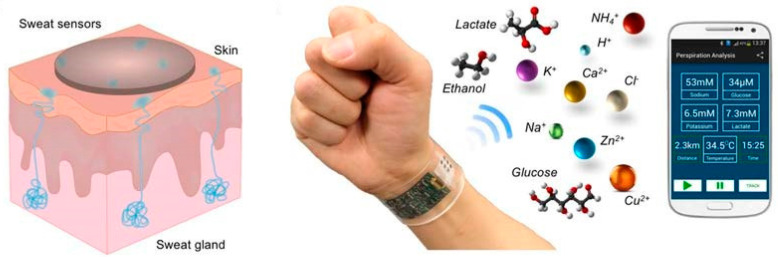
Wearable sweat biosensors which continuously measure a variety of sweat components for health monitoring. Reproduced from [164] with permission from IEEE.

For a more practical use in health status monitoring, different working groups developed multiplex analysis platforms. One interesting approach was to measure simultaneously glucose, lactate, sodium, potassium, and skin temperature in one fully integrated sensor array [137]. The patch-type sensor was flexible, wearable, and made of a PET substrate (Figure 7). The metabolites were detected by using GOx and LOx which were imbedded in a chitosan film (amperometric read out). Moreover, the analysis of sodium and potassium were realized by integrating ion selective electrodes (potentiometric read out). The skin temperature measurement was based on a chromium/gold metal microwire. Additionally, the electronic parts were sealed and covered with insulating parylene.

Another approach was made for the simultaneous detection of ethanol, glucose, and lactate with a low sample volume of sweat based on non-faradaic chronoamperometric read out [165]. For this purpose, nanotextured zinc oxide films were integrated on a flexible porous membrane. The specific enzymes were immobilized in the active zinc oxide region by using a linker molecule (DSP). A streptavidin biotinylated AOx was used for the detection of alcohol, whereas a glucose antibody and coupled GOx, and LOx was used for glucose and lactate, respectively. The measured changes in current were associated with interactions of the target biomarkers with their specific enzyme and the relating analyte.

### 2.4. Biosensors Operating in Urine

Urine, a typically sterile liquid by-product, is often used as a diagnostic tool for many disease conditions. An adult human produces around two liters of urine per day within about seven urinations. The number of urinations depends on state of hydration, activity level, environmental factors, weight, and the individual’s health. About 95% of the urine consists of water, but the fluid also contains different inorganic and organic, low and high molecular weight compounds [166]. A variety of compounds with clinical relevance are present in urine, such as glucose, lactate, urate, ascorbate, cholesterol and oxalate, all of which can be converted by different oxidoreductases. The occurrence of glucose in urine is associated with diabetes [167,168], whereas lactate is a prognostic marker for various disorders, and urinary lactate have been shown to correlate with blood lactate [169]. Significant amounts of cholesterol were detected in the urine of nephritic patients, whereas renal excretion of the bioanalyte is unsubstantial in healthy individuals [170]. Additionally, there are reports showing correlations between other biomarker levels in blood/plasma and urine, e.g., ascorbate and oxalate. For instance, oxalate concentrations in regular urine and blood range from about 160 µM to about 550 µM [171], and from about 17 µM to about 39 µM, respectively [172]. Ascorbate concentrations in plasma and urine of apparently healthy volunteers were found to be 76.50 ± 8.88 µM and 5.94 ± 1.43 µM, respectively [173]. Moreover, it was shown that increased renal excretion of ascorbate because of certain illnesses, e.g., sickle cell anaemia, might result in decreased plasma levels.

**Figure 8:**
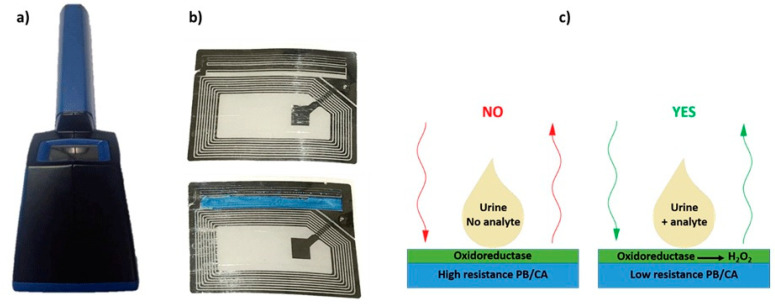
(**a**) Photographs of the portable monitoring unit, (**b**) sensor circuit developed for moisture detection (top) and modified sensor circuit modified with PB/CA layer (bottom), (**c**) Schematics of wireless biosensing. Reproduced from [176] with permission from IEEE.

Biosensors based on oxidases are one of the well-known biosensors for the detection of bioanalytes in urine. These sensors detect the H_2_O_2_ generated, which can be used to estimate the concentration of the particular analyte [174]. The most common enzyme used for the detection of glucose in urine is, as in other cases, GOx [175]. One of the methods used for the detection of glucose in urine using amperometric biosensors is exploitation of conductometric biotransducer, which gives a binary response, when the analyte is present in urine. The working principle is based on a Prussian blue-cellulose acetate layer modified with GOx. When the substrate is present, H_2_O_2_ is formed, reacting with the layer (Figure 8). The reaction leads to the change of conductivity of Prussian blue-cellulose acetate layer making it possible to estimate the presence of glucose using a wireless biosensor [176]. Another reported method used for determination of glucose in urine is based on amperometry by using redox mediators and a bi-enzyme system. The measurement can be achieved by compressing electrically conductive carbon with the strip of a biosensor simultaneously having two redox mediators, i.e., an enzyme system for the oxidation of glucose, and silver/silver chloride reference electrode. The analysis readout can be achieved by applying a drop of urine on a sensor, where the result is compared with a standard calibration curve or by converting the current flow to some units of urine glucose levels [177].

Another important bioanalyte present in urine is urea. The analysis of urea in urine is mostly based on the measurement of NH^4+^ and HCO^3−^, which are the hydrolysis products. For detection, potentiometric, amperometric, optical, thermal, piezo-electric, and conductometric sensors are used [178]. The most reliable sensor for measuring urea in urine is the amperometric urease biosensor, which is relatively simple and offers a low cost analysis. Results obtained with this sensor are directly associated with hydrolysis of urea on the electrode surface [179]. One of the first potentiometric urea sensitive biosensors designed in 1969, was also based on urease [180].

**Figure 9:**
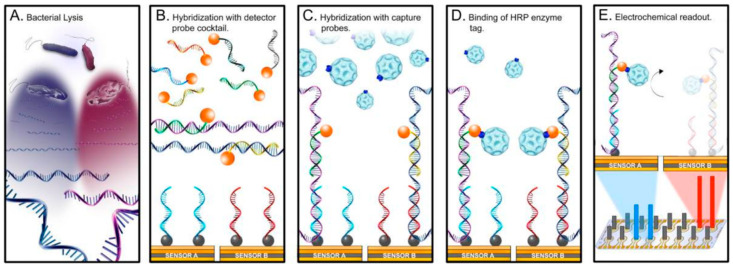
Multiplex pathogen detection scheme using an array of 16 electrochemical biosensors (UTI sensor array). Reproduced from Reproduced from [181] with permission from the US National Library of Medicine.

One of the most common bacterial infections, which poses a significant healthcare problem, is urinary tract infection (UTI). The standard culture-based diagnosis of UTI has a typical lead time of several days, and in the absence of microbiological diagnosis at point of care facilities, physicians frequently initiate broad-spectrum antibiotic treatment, thus contributing to the emergence of resistant pathogens. The powerful diagnostic platforms for infectious diseases are based on biosensors. For instance, an interesting example of a biosensor for uropathogen identification is the UTI Sensor Array (Figure 9) [181,182]. An electrochemical sensor array customized with bacteria specific DNA probes as recognition elements represents the sensor-platform. Each of the 16 sensors is modified with a self-assembled monolayer, which allows versatility in surface modification and, simultaneously, reduces background noise [183]. On the surface of the sensor, a library of DNA probes targeting the most common uropathogens is immobilized [184,185]. The detection protocol is based on conversion of hybridization events into quantifiable electrochemical signals.

Currently, biosensor diagnosis for UTI has moved beyond the proof-of-concept stage into the validation phase, with authentic clinical samples, and development of assays for rapid molecular pathogen identification and antimicrobial susceptibility testing. UTI sensor arrays offer a promising technology platform without the need for nucleic acid amplification. Removal of the technology bottlenecks still remaining, i.e., sample preparation and system integration, is crucial for the technology to be used in decentralized settings such as clinicians’ offices and emergency departments.

A novel innovative approach draws on a single platform strategy, i.e., a so-called universal electrode, that incorporates the central microfluidics of molecular analyses, i.e., pumping, mixing, washing, and sensing [186]. In an important illustration of the validity of the universal electrode platform, a bacterial phylogenetic marker was detected, promoting the rapid diagnosis of urinary tract infections. Since the platform is operated with electronic interfaces only, not only will it be possible to streamline systems integration and thus unlock the potential of microfluidics in molecular diagnostics at point of care sites, but also offer advanced biosensing in uncustomary health care settings [186].

Last but not least, recent efforts have been directed towards the development of wireless bacteria sensitive biosensors based on near field communication and radio frequency identification tags [187,188]. The approaches were realized by coupling a biosensor electrode as a part of the tag antenna. The transduction mechanism of these wireless biosensors exploits biological redox reactions. Specifically, the reactions change the impedance of the tag antennas, which is then wirelessly monitored by vector network analyzer or mobile phone. Future development of these wireless biosensors tags will target monitoring of UTI, as well as monitoring of bacterial growth in hygiene and medical products.

## 3. Conclusions and Outlook

Utilizing biological fluids for health monitoring offers the opportunity for non-invasive measurements and straightforward sample collection [55,134,135,137,138,147]. However, for successful sensor development, full and detailed knowledge is needed of the biological and chemical characteristics of sweat, saliva, tears, and urine, as well as the required technology for sensor realization [54]. Therefore, further efforts and research are needed to recognize the full diagnostic potential, in order to bypass the remaining challenges regarding sample collection, measurement, and sensing [147]. As an example, controlled and reproducible sampling is essential to improve the reliability of the results [54]. As regards sweat and tears, sample collection methods are wanting, and separate collection and analysis stages are in use [23,47]. Moreover, perspiration needs to be generated by exercising, heating, stress, or iontophoretic stimulation [54]. Additionally, variations in environmental and personal conditions, like temperature or pH, the individual skin composition, state of the oral cavity, or type of collected tears, fluid contamination, or mixing old and new fluid during sensing/sample collection impede reliable results [69]. Other challenges thwart the improvement of sensor characteristics, e.g., stability, biofouling, sensitivity, selectivity, robustness, accuracy, and power supply [24]. For instance, physiological fluids are complex solutions, provoking the integrity of the working electrode [142], and stability enhancing measures need to be taken in order achieve long term, continuous monitoring and measurement [54]. Moreover, electrodes should be usable without pre-treatment [24] or calibration, or storage in conditioning solutions (ion selective electrodes) [23,24]. On a final note, the power supply and packaging is one of the challenges for using sensors with an electro(-chemical) read out. Additionally, the packaging and integrated electronics should be in one comfortable, reliable, and safe platform [24].

After this presentation of a somewhat motley collection of vehicles/fluids, analytes, sampling techniques, sensors and sensor technology, power supplies, communication and data processing, it should be apparent to the reader that the field of non-invasive sensing of biomarkers in bodily fluid is highly convoluted. Nonetheless, if the drawbacks are appropriately addressed, and the pitfalls are adroitly circumvented, the approach will most certainly disrupt current clinical and self-monitoring practices.

As an example, the formation rate of all four fluids, affecting the availability of analytes, is varying in ways that are difficult to appreciate and control. However, by adhering to strict sampling protocols, relevant to clinical or point-of-care settings, or by relying on continuous measurements over an extended time, relevant to self-monitoring settings, much of the uncertainty emanating from varying fluid formation rates can be removed. Analogously, the blood concentration of some important biomarker targets is not exactly mirrored in the fluids under investigation. Depending on the particular target, the blood/fluid concentration discrepancy can be temporal or permanent. If the real (i.e., blood) value is critically needed, individual benchmarking using blood analysis combined with long term data collection usually featured by non-invasive sensors can be employed. If, on the other hand, the real value is non-critical, the aforementioned data collection features can identify positive or negative trends or abrupt baseline changes.
